# Investigating the factors influencing antibiotic use practices and their association with antimicrobial resistance awareness among poultry farmers in Enugu State, Nigeria

**DOI:** 10.1017/ash.2025.10141

**Published:** 2025-09-25

**Authors:** Chika P. Ejikeugwu, Emmanuel A. Nwakaeze, Chikaodi W. Aniekwe, Euslar N. Onu, Michael U. Adikwu, Peter M. Eze

**Affiliations:** 1 Department of Pharmaceutical Microbiology and Biotechnology, Enugu State University of Science and Technology (ESUT), Agbani, Nigeria; 2 Department Angewandte Mikrobielle Ökologie, Helmholtz-Zentrum für Umweltforschung – UFZ, Leipzig, Germany; 3 Department of Pharmaceutical Microbiology and Biotechnology, Chukwuemeka Odumegwu Ojukwu University, Igbariam, Nigeria; 4 Department of Microbiology, Faculty of Biological Science, Alex Ekwueme Federal University Ndufu-Alike, Ikwo, Nigeria; 5 Department of Pharmaceutics, University of Nigeria Nsukka, Enugu, Nigeria; 6 Department of Environmental Health Science, Nnamdi Azikiwe University, Awka, Nigeria; 7 School of Biological Sciences, Queen’s University Belfast, Belfast, NI, UK; 8 Department of Agriculture and Food, University of La Rioja, Logroño, Spain

## Abstract

**Objective:**

Effectively addressing poultry farmers’ antibiotic use and its role in antimicrobial resistance (AMR) presents a significant challenge, but improving their knowledge and practices is crucial for mitigating AMR risks and safeguarding public health. This study aimed to assess farmers’ understanding and behaviors to identify public health risks and inform targeted interventions.

**Design:**

Survey-based cross-sectional study.

**Setting:**

200 poultry farms in Enugu State, Nigeria.

**Participants:**

Poultry farmers.

**Methods:**

A cross-sectional survey of 200 poultry farms in Enugu State, Nigeria, was conducted using a validated questionnaire targeting farmers responsible for key farm decisions. The questionnaire covered sociodemographic data, AMR knowledge, and antibiotic use practices. Ethical approval was obtained, and participants provided oral consent.

**Results:**

Findings showed that 90.5% of farmers used antibiotics, primarily for treating infections (80.5%) and for growth promotion or prophylaxis (61%). Ampicillin (75%), ciprofloxacin (71.5%), and doxycycline (71%) were the most commonly used antibiotics, with monthly administration being prevalent (48%). Additionally, 89% of respondents believed antibiotics promote poultry growth. Alarmingly, 65% were unaware of AMR, and only 16% recognized its health risks.

**Conclusion:**

The heavy reliance on antibiotics, particularly ampicillin, raises concerns about beta-lactamase selection amid Nigeria’s carbapenem resistance issues. The significant knowledge gap among farmers highlights the urgent need for targeted education and stricter antibiotic regulation to mitigate AMR risks in poultry farming.

## Introduction

Antimicrobial resistance (AMR) is a major global public health threat, driven largely by antibiotic misuse in animal production, particularly poultry farming.^1,2^ The persistence of multidrug-resistant bacteria, including carbapenemase producers (eg, metallo-beta-lactamases), in Nigeria highlights the urgent need for intervention.^1,2^ Reducing antibiotic use in livestock could mitigate AMR spread, but addressing ecological gaps and improving knowledge of antibiotic use in food production is crucial. Studies indicate that antibiotic use in food animals significantly contributes to AMR emergence, resistant bacteria, and antibiotic resistance genes (ARGs) in the environment.^1–4^ Globally, around 75% of antibiotics are used in livestock, often as growth promoters or for disease prevention.^4–6^ Projections estimate that by 2,050, AMR could cause 10 million deaths annually.^5^ Developing countries, including Nigeria, face a rising AMR burden due to unregulated antibiotic use in poultry farming.^1,7^ Poultry farms in Nigeria serve as a major source of environmental antibiotic contamination, particularly through soil pollution, where the resistome facilitates the evolution and spread of resistance genes.^6–9^ Over-the-counter antibiotic sales without prescriptions are common,^1,10^ and many poultry farmers lack formal training, leading to antibiotic misuse.^11–13^ Antibiotics are often administered without veterinary guidance, and failure to observe withdrawal periods before slaughter increases the risk of residues and resistant bacteria entering the food chain. Consequently, poultry farms act as reservoirs for AMR dissemination.^1,10^ Although studies have examined AMR prevalence in Nigerian poultry production,^14^ limited research exists on farmers’ perspectives regarding antibiotic use and resistance, particularly in Enugu State. Poultry manure, commonly used as fertilizer, further exacerbates environmental contamination, contributing to resistant bacteria spread through runoff and leaching into water systems.^6–9^ Understanding farmers’ knowledge, attitudes, and practices (KAP) is vital for informing strategies to curb antibiotic misuse and AMR spread in Southeast Nigeria. Given poultry farming’s economic importance, assessing farmers’ knowledge and behavior is essential for safeguarding antibiotic efficacy and public health. Research suggests that farmer education significantly influences antibiotic use decisions.^10^ Targeted training programs could foster responsible antibiotic use and reduce AMR in poultry systems.^11,12^ This study aimed to investigate the drivers of irrational antibiotic use in poultry farming, which fuel AMR in the environment. A validated questionnaire collected data on farmers’ sociodemographics, AMR knowledge, and antibiotic use practices. Face-to-face interviews minimized bias. The findings will bridge knowledge gaps by assessing KAP among poultry farmers in Enugu State and contribute to mitigating public health risks associated with AMR.

## Materials and methods

### Study design, location, and sampling approach

**Figure 1.**
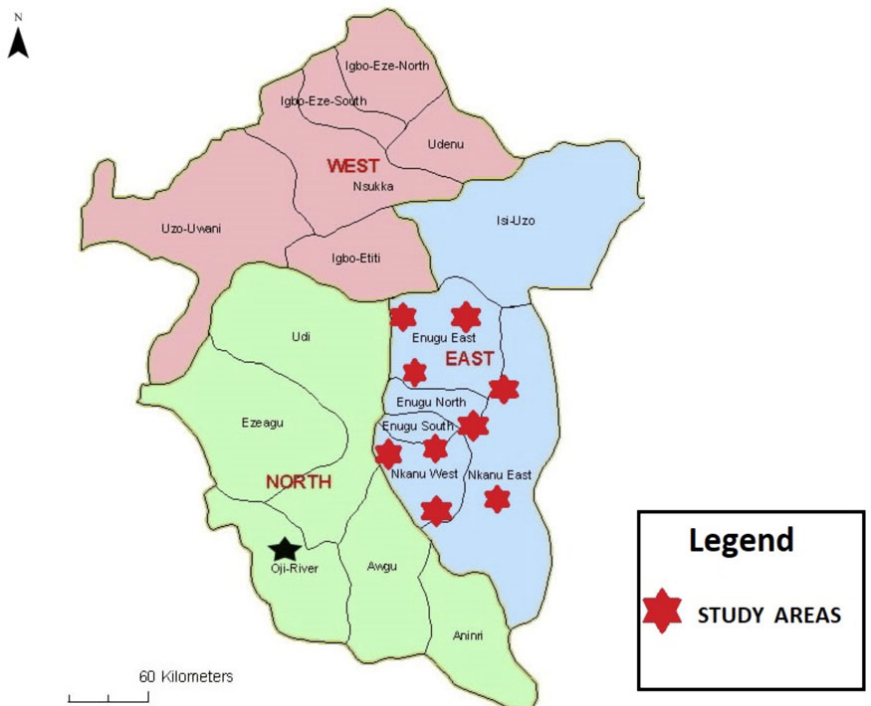
Map of Enugu State, Nigeria, showing study locations (map adapted from *Enugu Connect).*^15^.

The study was conducted from April 1 to August 31, 2024, using a cross-sectional design. A total of 200 poultry farms, each with at least 100 birds, were selected through stratified random sampling to ensure representation across the major geographic zones of Enugu State, Nigeria (Figure 1). Within each zone, farms were randomly selected from updated lists provided by local veterinary offices and poultry associations. The study focused on poultry farmers directly responsible for key agricultural decisions, including antibiotic use, feed management, and input procurement.

### Study instrument

A standardized, self-administered questionnaire in English was used to collect data on sociodemographics, AMR knowledge, and antibiotic use practices. Adapted from validated tools used in similar studies in Nigeria and other low- and middle-income countries (LMICs),^12–14^ it was reviewed by veterinary and epidemiology experts for content validity. A pilot test with 10 poultry farmers (excluded from the main study) assessed clarity and reliability, informing minor revisions. The final questionnaire had three sections: (1) sociodemographics; (2) AMR knowledge; and (3) antibiotic use practices. Pretesting with a veterinarian ensured accuracy. Responses were collected anonymously in categorical, ordinal, and open-ended formats for both quantitative and qualitative analysis.

### Data management and analysis

Data were collated and analyzed using Microsoft Excel version 16.92. Categorical variables were summarized as frequencies and percentages to provide insights into prevalent farming practices, as well as the respondents’ knowledge and awareness levels concerning AMR and antibiotic use. A two-way ANOVA was performed to evaluate statistical significance across the respondent outcomes.

### Ethical approval

Ethical approval for the study was granted by the Enugu State Ministry of Health (Reference number: MH/MSD/REC21/630). Informed oral consent was obtained from all study participants. Additionally, participants provided consent for the publication of the study data.

## Results

### Sociodemographic features of poultry farmers

This study included 200 privately owned poultry farms (each with at least 100 birds) in Enugu, Southeast Nigeria. Table 1 presents sociodemographic characteristics (gender, age, marital status, occupation, education, and internet usage) influencing farmers’ KAP regarding antibiotic use and AMR. Female respondents (56%) slightly outnumbered males (44%). Most farmers (52%) were aged 21–40 years, while younger (<20 yr, 4%) and older (>60 yr, 11.5%) groups were minimally represented (Table 1). No significant associations were found between knowledge of antibiotic use and sociodemographic factors (*p* > 0.05; Table S1). Male (93.18%) and female (97.32%) respondents showed high knowledge (*p* = 0.570). Across age groups, knowledge ranged from 90.91% (41–50 yr) to 100% (*p* = 0.392). Marital status (*p* = 0.487), occupation (*p* = 0.448), and education (*p* = 0.457) had no significant impact. University graduates (38%) were the largest group; primary school graduates were minimal (2.5%). Daily internet use was 54%, with no significant effect on knowledge (*p* = 0.467). Overall, poultry farmers exhibited strong awareness of antibiotic use and AMR (Table S1).

**Table 1.** Distribution of the studied respondents according to socio-demographic data

Demographics	Variable	*n*	%
Gender	Male	88	44
	Female	112	56
	**Total**	200	100
Age	<20 years	8	4
	21–30 years	54	27
	31–40 years	50	25
	41–50 years	44	22
	51–60 years	21	10.5
	>60 years	23	11.5
	**Total**	200	100
Marital status	Single	66	33
	Married	132	66
	Divorced	1	0.5
	No response	1	0.5
	**Total**	200	100
Occupational status	Employed	74	37
	Self Employed	42	21
	Housewife	16	8
	Househusband	1	0.5
	Student	36	18
	Retired	16	8
	Others	14	7
	No response	1	0.5
	**Total**	200	100
Highest academic qualification	Primary	5	2.5
	Secondary School	62	31
	University	76	38
	Diploma	12	6
	OND/HND	11	5.5
	Masters	18	9
	Doctorate	8	4
	No response	8	4
	**Total**	200	100
Internet usage	Everyday	108	54
	Often	57	28.5
	Rarely	21	10.5
	Never	13	6.5
	No response	1	0.5
	**Total**	200	100

### KAP of poultry farmers regarding antibiotic application in poultry farms

**Figure 2.**
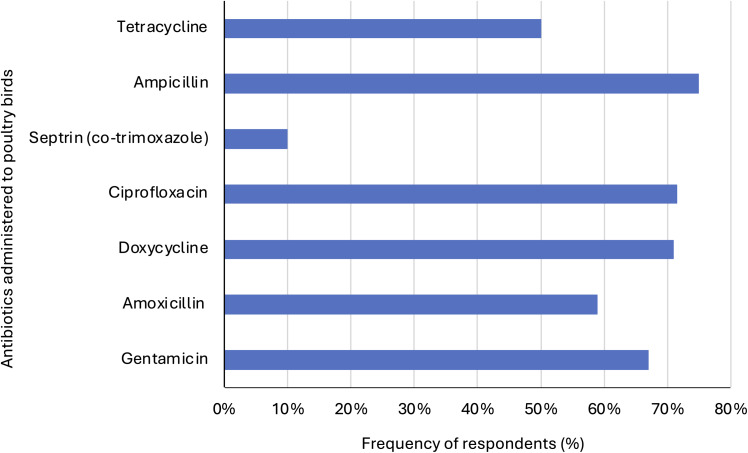
Commonly used antibiotics in poultry farms in Enugu, Nigeria.

About 80% of respondents knew the class, names, or brands of antibiotics used on their birds. While 2.5% applied antibiotics at all times, 24% administered them without symptoms, and 77% did so when signs of infection appeared. Only 15% followed veterinary recommendations (Table S2). Regarding frequency, 48% used antibiotics monthly, while 29.5% applied them bi-weekly. Ampicillin (75%) was the most used, followed by ciprofloxacin (71.5%), gentamicin (67%), and amoxicillin (59%). Most (80.5%) used antibiotics for treatment rather than growth promotion (12.5%), while 43.5% applied them prophylactically. Surprisingly, 89% believed antibiotics promote growth in poultry (Figure 2, Table S2). Regarding knowledge, 90.5% had used antibiotics in the past year. Only 42% considered vaccination a viable alternative, while 50.5% were uncertain. Notably, 86.5% consulted veterinarians before use (Figure 2, Table S2).

### KAP of poultry farmers regarding AMR

**Figure 3.**
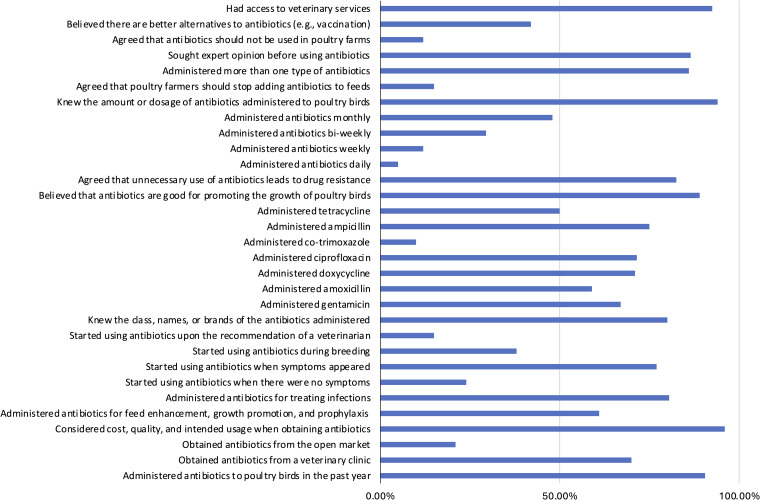
Distribution of insights into the KAP on antibiotics use by farmers in Enugu, Nigeria.

**Figure 4.**
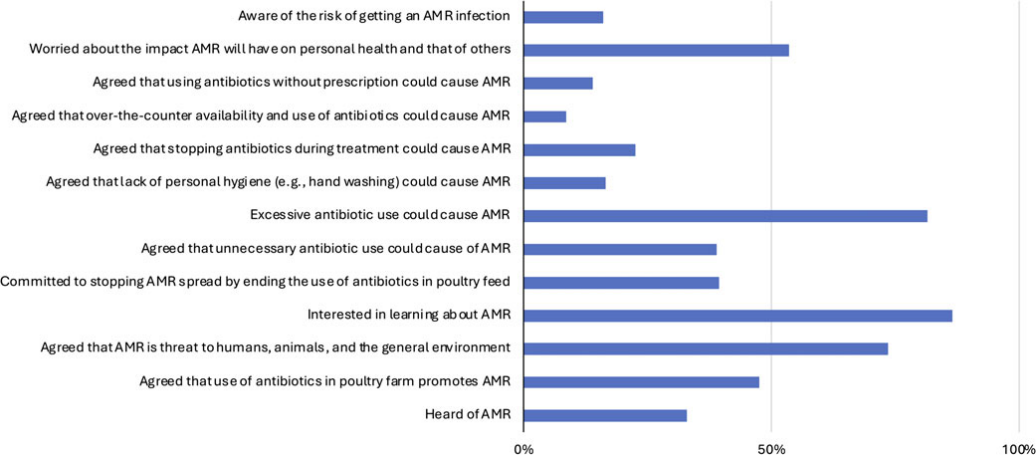
Distribution of insights into KAP on AMR by farmers in Enugu, Nigeria.

The poultry farmers’ KAP on AMR are summarized in Figure 3. Most (65%) were unaware of AMR, while 33% indicated awareness. Regarding antibiotics’ role in resistance, 42.5% agreed, 22.5% disagreed, and 25% were uncertain (Figure 4; Table S3). About 55.5% saw AMR as a health threat, but only 18% strongly agreed. Despite 86.5% wanting AMR education, only 39.5% were willing to stop antibiotic use in feed. Excessive use (81.5%), unnecessary use (39%), and treatment interruption (22.5%) were identified as major AMR contributors. Awareness varied by age (*p* ≤ 0.05), occupation (*p* = 0.006), education (*p* = 0.020), and internet use (*p* = 0.014) (Figure 4; Tables S3, S4).

## Discussion

AMR is rising globally, posing a serious health challenge. In Nigeria, poultry farming lacks standardized antibiotic guidelines, and over-the-counter (OTC) access drives unregulated use. This study evaluated poultry farmers’ KAP regarding antibiotic use and AMR in Enugu.

LMICs are disproportionately affected by AMR. Inadequate training and misuse of antibiotics in poultry farming remain key drivers. The use of antibiotics that are critical for human medicine, often without veterinary oversight, poses serious risks to both human and environmental health. In this study, 90.5% of poultry farmers used antibiotics, mostly for treatment, but 89% also reported use for growth promotion - a practice similarly reported in India and Pakistan.^16,17^ This suggests entrenched practices influenced by economic motivations and perceived benefits, despite growing global discouragement of antibiotic use for non-therapeutic purposes.

The prevalent belief (89%) that antibiotics promote growth underscores persistent misinformation and a gap in veterinary extension services. Commonly used antibiotics – ampicillin (75%), ciprofloxacin (71.5%), and doxycycline (71%) – are medically important, raising concerns over selective pressure and increasing resistance, including carbapenem resistance.^1,18^ Similar patterns in Southwest Nigeria and Pakistan^17,19^ reflect a regional trend of misuse. Widespread use of antibiotics from major therapeutic classes – including beta-lactams, fluoroquinolones, aminoglycosides, sulfonamides, and tetracyclines – in other parts of Nigeria^22^ underscores the broader national implications.

Although 86.5% of farmers sought expert advice, antibiotics were still widely sourced from veterinary clinics (70%), pharmacies (23%), open markets (21%), and patent medicine shops (14.5%). Easy access without stringent regulation facilitates self-medication and undermines rational use. Studies from Ethiopia, Burkina Faso, and Pakistan report similar dynamics.^14,17,19,21^ These patterns highlight a systemic weakness: insufficient regulation and monitoring of antibiotic sales and usage, alongside gaps in veterinary infrastructure.

Knowledge of AMR was low, with only 33% having heard of it. This aligns with findings from Reyher et al.^23^ and another study in Burkina Faso,^22^ where the majority of farmers lacked AMR awareness. Limited access to accurate information, reliance on informal networks, and inadequate farmer education contribute to this knowledge gap. Such systemic deficiencies create behavioral inertia, where farmers continue antibiotic use unaware of long-term risks.

Despite general awareness of AMR, misconceptions remain prevalent. Only 47.5% of respondents recognized the link between antibiotic use in poultry and AMR, while the rest were unsure or unaware, limiting the potential for behavior change. Younger, more educated farmers with internet access demonstrated greater awareness, with education, occupation, and gender significantly influencing AMR knowledge. Internet use was significantly associated with AMR awareness (*P* ≤ 0.05), underscoring the role of online information access. However, no significant association was found between internet use and antibiotic use practices (*P* > 0.05), suggesting that awareness alone does not translate into more responsible antibiotic use.

These insights point to key behavioral and systemic barriers – economic dependency on antibiotics, limited veterinary support, weak regulatory enforcement, and low risk perception. Tackling AMR requires a multifaceted strategy. Promoting non-pharmaceutical infection control – biosecurity, hygiene, and vaccination – is vital to reducing reliance on antibiotics.

Tailored educational campaigns addressing specific misconceptions, improving veterinary outreach, and enforcing OTC regulations are essential in settings like Nigeria. Although this study focused on Enugu, the findings reflect broader national trends and underscore the urgency of comprehensive antibiotic stewardship in poultry farming. As a next step, we aim to use whole-genome sequencing (WGS) to characterize the ARGs prevalent in these farms following antibiotic application, providing deeper insight into resistance mechanisms and environmental dissemination pathways.

This study offers context-specific insights into antibiotic use and AMR awareness among poultry farmers in Enugu, Nigeria identifying key misuse drivers to guide interventions. Study limitations include potential social desirability bias related to literacy and awareness. Future work will therefore stratify participants by education level and incorporate observational measures to mitigate this bias.

## Conclusion

AMR in Nigerian poultry farming is driven by antibiotic misuse, low awareness (33%), and unregulated access. About 90.5% of farmers use antibiotics, mainly for infections and growth. Ampicillin is the most used, raising beta-lactamase concerns. Urgent education, stricter regulations, and alternative practices are needed to curb AMR and protect public health.

## Supporting information

10.1017/ash.2025.10141.sm001 Ejikeugwu et al. supplementary material 1 Ejikeugwu et al. supplementary material

10.1017/ash.2025.10141.sm002 Ejikeugwu et al. supplementary material 2 Ejikeugwu et al. supplementary material

## Supplementary material

The supplementary material for this article can be found at https://doi.org/10.1017/ash.2025.10141.

## Transparency declarations

The authors declare no financial conflicts of interest related to this study. This research was funded by the National Institutes of Health (NIH) under the CAMRA (Combatting AntiMicrobial Resistance in Africa Using Data Science) initiative, Federal Award Number: 5U54TW012056-03. The funders had no role in the study design, data collection, analysis, manuscript preparation, or decision to publish. No professional medical writer or similar service was involved in the preparation of this manuscript. All authors have no conflicts of interest to declare.
