# Supplementary material for: Investigating the factors influencing antibiotic use practices and their association with antimicrobial resistance awareness among poultry farmers in Enugu State, Nigeria
Source: Antimicrob Steward Healthc Epidemiol. 2025 Sep 25;5(1):e236. doi: 10.1017/ash.2025.10141 (PMC12509157; doi:10.1017/ash.2025.10141)
Supplement: Ejikeugwu et al. supplementary material 1 — Ejikeugwu et al. supplementary material [file S2732494X25101411sup001.docx]

**Acknowledgment**

We acknowledge the management of Enugu State University of Science and Technology (ESUT), Agbani, Nigeria and the Administrative/Technical staff from ACEGID, Redeemer’s University, Ede, Osun State, Nigeria for all administrative support towards the successful conduct and completion of this study.

**Conflict of interest**

No conflict of interest exist among the authors

**Funding**

This study is part of the research project “Genomics to Monitor Abundances and Diversity of Antimicrobial Resistance (AMR) Genes and Strains Circulating in the Poultry Food Chain in Nigeria.” The project received funding from the National Institutes of Health (NIH) under the CAMRA (Combatting AntiMicrobial Resistance in Africa Using Data Science) initiative, Federal Award Number: 5U54TW012056-03. The authors extend their gratitude to the management of ESUT, Nigeria, for their administrative support.

**Supplementary Material**

The following Tables – S1: Relationship between respondents’ knowledge about antibiotic use and their socio-demographic data; Table S2: Knowledge, Attitude and Practice on Antibiotics Use; Table S3: Knowledge, Attitude and Practice on Antimicrobial Resistance (AMR); and Table S4: Relationship between respondents’ knowledge about AMR and their socio-demographic data – are available in in the supplementary material.

**Authors contribution**

Conceptualization: CPE

Methodology: CPE, CWA, PME

Formal analysis: EAN, ENO, MUA, PME

Data analysis: CPE, EAN, PME

Supervision: CPE, MUA, PME

Writing, review and editing: CPE, EAN, CWA, ENO, MUA, PME

**Transparency declarations**

The authors declare no financial conflicts of interest related to this study. This research was funded by the National Institutes of Health (NIH) under the CAMRA (Combatting AntiMicrobial Resistance in Africa Using Data Science) initiative, Federal Award Number: 5U54TW012056-03. The funders had no role in the study design, data collection, analysis, manuscript preparation, or decision to publish. No professional medical writer or similar service was involved in the preparation of this manuscript. All authors have no conflicts of interest to declare.

**Word count: 37,447**
